# Correction: A multi-stage process to develop quality indicators for community-based palliative care using interRAI data

**DOI:** 10.1371/journal.pone.0316372

**Published:** 2024-12-19

**Authors:** Dawn M. Guthrie, Nicole Williams, Cheryl Beach, Emma Buzath, Joachim Cohen, Anja Declercq, Kathryn Fisher, Brant E. Fries, Donna Goodridge, Kirsten Hermans, John P. Hirdes, Hsien Seow, Maria Silveira, Aynharan Sinnarajah, Susan Stevens, Peter Tanuseputro, Deanne Taylor, Christina Vadeboncoeur, Tracy Lynn Wityk Martin

The 19^th^ author’s name is spelled incorrectly. The correct name is: Tracy Lynn Wityk Martin.

S1 File is uploaded incorrectly. Please view the correct S1 File below.

## Supporting information

S1 File (DOCX)

## References

[1] Guthrie DM, Williams N, Beach C, Buzath E, Cohen J, Declercq A, et al. (2022) A multi-stage process to develop quality indicators for community-based palliative care using interRAI data. PLOS ONE 17(4): e0266569. doi: 10.1371/journal.pone.0266569 35390091 PMC8989210

