# Supplementary material for: A multi-stage process to develop quality indicators for community-based palliative care using interRAI data
Source: PLoS One. 2022 Apr 7;17(4):e0266569. doi: 10.1371/journal.pone.0266569 (PMC8989210; doi:10.1371/journal.pone.0266569)
Supplement: S1 Table — (DOCX) [file pone.0266569.s001.docx]

**Operational definitions for each of the 27 quality indicators (QIs)**

| **Quality Indicator** | **Operational definitions of numerator and denominator** |
| --- | --- |
| Prevalence of falls | Numerator: Client experiences a fall within the last 90 days  Denominator: All clients not completely dependent in bed mobility on previous assessment |
| Prevalence of severe or excruciating daily pain | Numerator: Client experiences daily pain AND pain is severe or excruciating  Denominator: All clients on re-assessment |
| Prevalence of pain that is not controlled by medications | Numerator: Client experiences pain AND pain is not controlled by medications  Denominator: All clients that have pain on re-assessment |
| Failure for pain to improve | Numerator: Client has pain AND pain does not improve on re-assessment  Denominator: All clients with at least one re-assessment AND had pain at previous assessment |
| Prevalence of constipation | Numerator: Client experiences constipation - no bowel movement in 3 days or difficult passage of hard stool  Denominator: All clients on re-assessment |
| Prevalence of shortness of breath at rest | Numerator: Client experiences shortness of breath at rest  Denominator: All clients on re-assessment |
| Prevalence of shortness of breath upon exertion | Numerator: Client experiences shortness of breath when performing normal day-to-day activities  Denominator: All clients on re-assessment |
| Failure for shortness of breath to improve | Numerator: Client experiences shortness of breath (at rest or upon exertion) on two consecutive assessments  Denominator: All clients with at least one re-assessment AND had shortness of breath at previous assessment |
| Prevalence of stasis/pressure ulcers | Numerator: Client experiences a pressure OR stasis ulcer  Denominator: All clients on re-assessment who are not imminently dying (death expected within days) |
| Prevalence of a delirium-like syndrome | Numerator: Client experiences an acute change in mental status from their usual functioning and they experience one of: a fluctuating state of consciousness, mental functioning that varies over the course of the day, hallucinations or delusions  Denominator: All clients on re-assessment |
| Prevalence of nausea or vomiting | Numerator: Client experiences nausea or vomiting  Denominator: All clients on re-assessment |
| Prevalence of fatigue | Numerator: Client has the inability to complete normal daily activities (e.g., ADLs, IADLS) due to diminished energy  Denominator: All clients on re-assessment |
| Prevalence of sleep problems | Numerator: Client has difficulty falling asleep, staying asleep, waking up too early, experiences restlessness or experiences non-restful sleep  Denominator: All clients on re-assessment |
| Prevalence of poor self-reported health | Numerator: Client reports their health as being fair or poor  Denominator: All clients on re-assessment |
| Prevalence of negative mood | Numerator: Client has a Depression Rating Scale (DRS) score of ≥4  Denominator: All clients on re-assessment |
| Failure for negative mood to improve | Numerator: Client has a DRS score ≥4 and does not improve on re- assessment  Denominator: All clients with at least one re-assessment AND DRS ≥4 on previous assessment |
| Prevalence of declining social activities that causes distress | Numerator: Client experiences a decline in their social activities that causes them distress  Denominator: All clients on re-assessment |
| Prevalence of loneliness | Numerator: Client reports feeling lonely  Denominator: All clients on re-assessment |
| Prevalence of caregiver distress | Numerator: Client’s primary caregiver expresses feelings of distress, anger, or depression  Denominator: All clients on re-assessment with a primary caregiver |
| Prevalence of anxious complaints | Numerator: Client has repetitive anxious complaints/concerns  Denominator: All clients on re-assessment |
| Prevalence of struggling with meaning of life | Numerator: Client indicates that they are struggling with the meaning of life  Denominator: All clients on re-assessment |
| Prevalence of clients feeling a lack of completion of financial, legal and other formal responsibilities | Numerator: Client does not have a sense of completion on transfer of financial, legal and other formal responsibilities (e.g., power of attorney, will).  Denominator: All clients on re-assessment |
| Prevalence of clients feeling that progress is not being made regarding completion of personal goals | Numerator: Client does not have a sense of making progress regarding completion of personal goals  Denominator: All clients on re-assessment |
| Prevalence of wanting to die now | Numerator: Client expresses to friends, family members or staff that they want to die now  Denominator: All clients on re-assessment |
| Prevalence of emergency department visit | Numerator: Client experiences ≥ 1 emergency room visit(s) without an overnight stay within the last 90 days  Denominator: All clients on re-assessment who are not imminently dying (death expected within days) |
| Prevalence of hospital admission | Numerator: Client experiences ≥1 hospital admission(s) with an overnight stay within the last 90 days  Denominator: All clients on re-assessment who are not imminently dying (death expected within days) |
| Prevalence of no advance directives | Numerator: Client has no advance directives for avoiding common medical treatments (e.g., no directive for resuscitation nor for hospitalization)  Denominator: All clients on re-assessment |
