# Supplementary material for: A multi-stage process to develop quality indicators for community-based palliative care using interRAI data
Source: PLoS One. 2022 Apr 7;17(4):e0266569. doi: 10.1371/journal.pone.0266569 (PMC8989210; doi:10.1371/journal.pone.0266569)
Supplement: S1 File — (DOCX) [file pone.0266569.s002.docx]

**An example of applying the interpercentile range adjusted for asymmetry (IPRAS)**

The interpercentile (IPR) range adjusted for asymmetry (IPRAS) is a method to deal with the issue that the level of disagreement among panel members tends to increase as panel sizes get larger (e.g., beyond n=15). Since the current sample had 21 experts, we chose this method, which can be applied to any panel size. A hypothetical example is used here for explanation purposes.

***Example:*** In this example, we assume that we are looking at the ratings, across all 21 panel members, for the first evaluation criterion, namely “importance” of the QI. This process was applied similarly to all four evaluation criteria. We will assume the median score, across the 21 panel members, was 9.

- Median score=9
- Lower percentile for the IPR (30^th^ percentile)=8
- Upper percentile for the IPR (70^th^ percentile)=9
- Interpercentile range (IPR)= Upper percentile – Lower percentile=9-8=1
- IPRCP (IPR central point)= (Lower percentile + Upper percentile)/2 =(8+9)/2 =8.5
- Asymmetry index (AI)= 5-IPRCP=5-8.5=3.5

**The AI is expressed as an absolute value and is the difference between the central point of the IPRCP and the scale*

- Interpercentile range adjusted for asymmetry (IPRAS)= 2.32 + (1.5 * AI) =2.32 + (1.5*3.5)=7.6
- DI (disagreement index)= IPR/IPRAS =1/7.6 =0.13

Since the DI value is less than one, this criterion would be considered to have “agreement.” If the DI value was greater than or equal to one, the criterion would be defined as having “disagreement”[1].

**References**

1. Fitch K, Bernstein SJ, Aguilar MD, Burnand B, LaCalle JR, Lazaro P, et al. The RAND/UCLA appropriateness method user's manual. Santa Monica, CA: 2001.
