# Supplementary material for: A multi-stage process to develop quality indicators for community-based palliative care using interRAI data
Source: PLoS One. 2022 Apr 7;17(4):e0266569. doi: 10.1371/journal.pone.0266569 (PMC8989210; doi:10.1371/journal.pone.0266569)
Supplement: Attachment — Submitted filename: 2022-01-03_QI-palliative-care.docx [file pone.0266569.s003.docx]

Review comments on „A multi-stage process to develop quality indicators for community-based palliative care using interRAI data“

1. Line 344: The authors write that *“the proposed QIs provide a relatively comprehensive picture of the issues that are 345 widely accepted as key metrics when assessing the quality of PC services[61]*.” What does “comprehensive” mean in this context? I strongly suggest that you critically discuss the content validity not only of single indicators, but also of the indicator set as a whole (see e.g. Schang L, Blotenberg I, Boywitt D. What makes a good quality indicator set? A systematic review of criteria. Int J Qual Health Care. 2021 Jul 31;33(3):mzab107. doi: 10.1093/intqhc/mzab107. PMID: 34282841; PMCID: PMC8325455.)

Specifically, it would be helpful to explicitly report on the degree to which the proposed QI cover the 6 themes of palliative care that you mention at the beginning of your paper, the gaps in your indicator set and whether the proposed QI represent a balanced view of what is important for palliative care patients, e.g. with respect to the 6 themes.

1. Table 1: Why is the distinction between “clinical” and “psychosocial” indicators relevant? I would expect more content-oriented distinctions between indicators, e.g. with respect to the 6 themes of palliative care you mention.
2. While a Delphi study can help to capture judgements by experts, the qualitative reasons put forward for quantitative ratings remain a “black box”. Also, what does the following sentence mean: “It should be noted that the research team utilized the Delphi results as a guide, to support decision-making, but not as a constraint” (line 272 f.)? To enhance transparency of your findings, I strongly suggest reporting for each QI why you dropped or kept them, and how you made that decision.
3. Why did you decide to drop important indicators only because no data was available? If these indicators were important, wouldn’t it be valuable to develop the required data, e.g. expand on existing interRAI assessments?
4. It remains unclear whose quality is to be measured by the indicators – are specific providers accountable for the features measured? Or a regional health system as a whole? Please specify.
5. Please specify what RN (line 114) means – research nurse?
6. What exactly are “knowledge users” (line 160) and why is it important that they did the recruitment of patients and family members?
