# Supplementary material for: A multi-stage process to develop quality indicators for community-based palliative care using interRAI data
Source: PLoS One. 2022 Apr 7;17(4):e0266569. doi: 10.1371/journal.pone.0266569 (PMC8989210; doi:10.1371/journal.pone.0266569)
Supplement: Attachment — Submitted filename: response to reviewers_jan2622.docx [file pone.0266569.s004.docx]

**Comments from Academic Editor:**

1. In your data sharing statement you state that the secondary data are available by placing a request with the Canadian Institute for Health Information, and that the data do not belong to the researchers. Can you please clarify exactly what data that you used in this study is held with this organisation? It's currently unclear how you collected data from the Delphi panel participants, which you appeared to convene for the purpose of this study, without having control of the data. It's also unclear why this anonymised data could not be included with the submitted paper as per the journal's policy on data sharing.

*The data held by CIHI are based on the interRAI assessments, whose data elements were used to define the QIs. To be clear, we did not use the interRAI data in our analysis. In hindsight, we feel that perhaps this should not have been mentioned in the data sharing statement, and we have fixed this accordingly. The other data collected from the Delphi participants is readily available and can be shared publicly.*

2. Linked to this is your statement that consent was not obtained from participants because you only used secondary anonymised data. Please explain how were data collected for a very specific research project such as this, where you describe recruiting Delphi panel participants, without collecting primary data. To me this appears to be primary data collection that would require participant consent.

*Individual consent WAS obtained from the Delphi panel members so this was an error in our earlier submission. It has been revised in the manuscript (line 240-241) and in the online form.*

3. Finally, you need to provide further details about recruitment methods in the manuscript. Reviewers 2 and 3 both mention the lack of clarity relating to recruitment to some extent, and this needs significantly strengthening beyond what the reviewers have asked for. What were the characteristics of this 'group of graduate students'? Eg how many were there, what was their expertise, and how did they relate to the project / research team beyond this exercise? For the Delphi panel participants, how were they identified? Who recruited them? How many people were approached and declined to participate or did not respond to an invitation (you provide a response rate, but that's based on total panel members not the total number of people approached to be panel members)? Was there any potential for bias (based on their characteristics)?

*We have added significant detail to respond to this concern about a lack of information. For example, we have reported that there were 15-20 graduate students who provided feedback, all from the University of Waterloo. None of these students were part of our research team, and they simply represented a convenience sample of graduate students at a nearby university (see lines 236-239).*

*For the Delphi panel, we have added additional details to provide a more fulsome understanding of the participants and non-respondents (lines 285-292). We invited members of our research team (n=12) to take part in the Delphi panel. These individuals were also asked to forward the names of other possible experts that would be appropriate for the panel and this resulted in 21 additional names. A member of our research team, typically a paid research assistant, contacted each of these 33 individuals and 21 consented and took part in the Delphi panel. Among the 12 individuals who did not complete the Delphi panel, one person felt that they did not have the expertise to do the evaluations, while the remainder (n=11), did not respond after repeated emails. The 12 individuals who consented but did not complete the Delphi panel represented a mix of individuals with different backgrounds (e.g., research, nursing, medicine, social worker, health care management/administration), so we do not feel that there was a bias among those who participated and those who did not.*

**Reviewer #1:**

1. The Q.Is were explicit although no real comment was made about the lack of expert by experience or service user input here.What is apparent is the nature of the Q.Is that were dropped. Although these were dropped because of Q.I scores they were focused on psycho social elements. The others were all medical which could be expected however, there are close links between both these elements. I am sure you agree. I understand that this would be a focus of the next stage of research? However, having a rational for this and ensuring that this is a key consideration would have reflected your statement of intention to rectify this.

*Some clarification is required here. For example, NONE of the QIs were put into the “discard” category following the Delphi panel. The 5 QIs which were ultimately dropped had been categorized initially as “review”, based solely on the Delphi scoring. These 5 QIs were then discussed by the entire team (see lines 313-317), who made the decision to drop these QIs from further consideration. We recognize that it would be ideal to have a broad scope of psychosocial QIs included in our final list, and this is something we will address in later stages of the research when we are using existing interRAI data to evaluate each individual QI and making a decision about which QIs will be included in the final list (see lines 3699-402).*

2. As you say, there was only one family member involved and this could be considered a gap.

*Actually, there were 9 family members interviewed in our project and one person with lived experience (i.e., one person actively receiving palliative home care; see lines 166-167). We have mentioned in the discussion that our inability to include more individuals receiving PC was a limitation (lines 368-370).*

**Reviewer #2:**

1. The criteria the author used was slightly different from the criteria in the previous literature the author cited (Importance, Scientific acceptability, Usability, and Feasibility). Furthermore, generalizability of the indicator is also one topic of the criteria for QI. How about the generalizability of these indicators outside the interRAI network? Please discuss.

*Our criteria quite closely follow those recommended by Seow et al. for cancer care (see reference #59 in the manuscript). For example, they suggest importance and usability as key criteria, both of which were used by our team. They also recommend “scientific acceptability”, which includes 5 different criteria, two of which are validity and evidence of improved outcomes, both of which were criteria that we used. We did not, however, use feasibility as a criterion. Feasibility, in this context, evaluates the extent to which data are readily available with which to generate the QIs. Given that our QIs were all defined based on existing interRAI data elements, this criterion was not applicable in our work, as all of the proposed QIs would be feasible to calculate for an organization using the interRAI assessments. The decisions regarding which criteria to use were made in conjunction with team members with extensive experience in conducting Delphi panels.*

*It is possible that some of the QIs we proposed would be able to be generated with different types of electronic data records, so long as the necessary data elements were embedded within the electronic health record. For example, organizations routinely collecting details with respect to pain, shortness of breath, nausea/vomiting, etc. would potentially be able to generate similar QIs for these issues. However, in order to explore all 22 QIs, an organization would need to be using an interRAI assessment as part of routine, clinical practice. As a result, as we mention in the discussion (see lines 380-391), the full set of QIs is limited to the data elements embedded within in the interRAI instruments.*

2. Modified Delphi approach usually includes two or more rounds excluding item generation phase. Why did the author perform only one round? Please discuss.

*After the initial round of the Delphi panel, there were 7 QIs which were categorized as “review” and which required further consideration. We opted not to conduct another full-blown Delphi panel given the extensive time commitment this would involve, since it took nearly a year to collect the data in round one. We felt that another full year of effort was not warranted to receive feedback on this small set of QIs. Instead, we met with our entire team to discuss these QIs, recognizing that several members of our team had taken part in round one and would therefore bring that experience to the discussion.*

3. How to determine the domain in this study group? That information would also be useful for readers to capture the process of consensus development.

*It is unclear what the reviewer means here by “domain in this study group”. We require some further explanation in order to know how best to answer this question.*

4. The description about panel members were slightly complex to capture. The relationship between 33 Delphi panel members and 30 PCs and other stakeholders was unclear. Who were these 33 members?

*For the Delphi panel, we have added additional details to provide a more fulsome understanding of the participants and non-respondents (lines 285-292). We invited members of our research team (n=12) to take part in the Delphi panel. These individuals were also asked to forward the names of other possible experts that would be appropriate for the panel and this resulted in 21 additional names. As noted in Table 2, those who took part in the Delphi panel tended to be female (71.4%), with a mean age of 46.3 years and most had been working in palliative care for more than 10 years (66.7%).*

5. Disagreement and panel decision about QI seemed to be complex to understand.First, according to the Step 2 in this manuscript, three groups (“discard”, “retain”, or “review”) seemed not to be collectively exhaustive. How to deal with the indicator when the median value was between 7-9 and no agreement in STEP1?

*We understand that this process is quite complex. In the example proposed here by the reviewer, the criterion would go into the review category as the median was “another value” and there was disagreement. Note that the “review” group is defined as “the median was between 4-6* ***OR*** *the median was another value AND there was disagreement in Step 1” (lines 271-272).*

6. Next, in STEP3, if the criteria were considered exact two “review” and two “retain”, which category should the QI be in?

*This QI would fall into the “review” category since this category includes those QIs where 2 criteria were “retain” (this is the first rule for Step 3) and it also includes those QIs for which 2, 3 or 4 of the criteria were “review” (the second rule outlined in Step 3).*

7. Third, how to determine “DROP” or “KEEP” in the Table3? This process was not described in Method section.

*We opted to include this decision in the results section, and not the methods section, since it was impossible to know which category the QIs would go into until after the results of the Delphi panel were known. We have described our decision-making in lines 313-317..*

8. Finally, Table3 only included appropriateness score for each QI. Disagreement/agreement of each category is necessary to understand the panel decision of each QI precisely. It seemed that the words (“review”, “retain”, and “discard”) were used both for the four criteria and for the individual QI, which could make readers confuse

*We have updated Table 3 so that the results from Step 1 (namely, agreement or disagreement on each of the 4 criteria) have been noted. We have also reorganized the list of QIs to be in line with the revisions made to Table 1 (see our response to Question 2 from Reviewer #3). We opted to keep the wording similar between these different steps in the process to be in line with the literature on how to carry out and score a Delphi panel. We feel this is a benefit to readers who are familiar with this process. For those who are unfamiliar with this process, we feel that keeping the terminology similar would actually be less confusing then if completely different terms were used in the various steps. We recognize that this is a complex process and we have done our best to outline all of the various steps in clear manner.*

9. There is no data about the numerator and denominator of each QI. Furthermore, the detail explanation of each QI should be described.

*We have added in an additional supplementary table which has the operational definitions for the numerator and the denominator for each QI (S1 Table).*

10. In supplemental file, “Interpercentile range (IPR)= Upper IPR – Lower IPR” is correct? Upper limit IPR?

*We modified this line to read “Interpercentile range (IPR)= Upper IPR percentile – Lower percentile IPR=9-8=1”. We made some other small changes to this supplemental file to try and keep the wording as clear as possible and to follow similar wording from one step to the next. We hope these changes will make this document, and this process, easier to follow.*

**Reviewer #3:**

1. Line 344: The authors write that “the proposed QIs provide a relatively comprehensive picture of the issues that are 345 widely accepted as key metrics when assessing the quality of PC services[61].” What does “comprehensive” mean in this context? I strongly suggest that you critically discuss the content validity not only of single indicators, but also of the indicator set as a whole (see e.g. Schang L, Blotenberg I, Boywitt D. What makes a good quality indicator set? A systematic review of criteria. Int J Qual Health Care. 2021 Jul 31;33(3):mzab107. doi: 10.1093/intqhc/mzab107. PMID: 34282841; PMCID: PMC8325455.)

   Specifically, it would be helpful to explicitly report on the degree to which the proposed QI cover the 6 themes of palliative care that you mention at the beginning of your paper, the gaps in your indicator set and whether the proposed QI represent a balanced view of what is important for palliative care patients, e.g. with respect to the 6 themes.

*Thank you for sharing the work by Schang et al. We reviewed this paper carefully and have added some text to outline our impressions of the content validity of our SET of indicators. For example, we point out that in 5 of the 6 themes discussed in Phase I, we were able to develop at least 1 QI in each of those themes. However, we could not create any QIs related to theme 6, home as the preferred place of death, as there are no data elements in the interRAI tools to capture this. We also added, in the discussion, that we were much better able to assess the “symptom management” theme than the other themes, based solely on the limitations in the data elements captured in the interRAI assessments. We have also commented on the extent to which our QI list meets other criteria, such as costs of measurement, prioritization, redundancy, and stakeholder involvement (lines 346-371).*

2. Table 1: Why is the distinction between “clinical” and “psychosocial” indicators relevant? I would expect more content-oriented distinctions between indicators, e.g. with respect to the 6 themes of palliative care you mention.

*Our initial categorization into ‘clinical’ and ‘psychosocial indicators’ was simply a way to stratify the QIs into some type of broad groups. We agree that separating the QIs into the 6 themes that were raised during Phase I. This table has been modified significantly. Please note that we removed the “track changes” function in word for this table as the huge number of changes made to the text made it extremely difficult to read the information in the table.*

3. While a Delphi study can help to capture judgements by experts, the qualitative reasons put forward for quantitative ratings remain a “black box”. Also, what does the following sentence mean: “It should be noted that the research team utilized the Delphi results as a guide, to support decision-making, but not as a constraint” (line 272 f.)? To enhance transparency of your findings, I strongly suggest reporting for each QI why you dropped or kept them, and how you made that decision.

*The quote cited here means that the Delphi results guided the decisions, but the research team also used their own discretion as to the final decisions regarding whether to keep a QI for further consideration or not. We have modified this statement to be more clear (line 279-280).*

*We have added further details to Table 3, as recommended by Reviewer #2 (see point 8).* *We modified the table so that the results from Step 1 (namely, agreement or disagreement on each of the 4 criteria) have been noted for each QI. This additional information, together with the panel decision in Table 3 summarizes these decision for most of the QIs. For the 7 QIs which the panel categorized as “review”, the team reviewed these and decided to drop 5 from further consideration (which is described on lines 311-315). We now feel that the decision-making process has been captured well with the information in Table 3, with the appendix defining how agreement/disagreement was determined (S2 file), and with some additional text in the results for 7 QIs which required further review.*

4. Why did you decide to drop important indicators only because no data was available? If these indicators were important, wouldn’t it be valuable to develop the required data, e.g. expand on existing interRAI assessments?

*We undertook this QI development work with the a priori interest in creating QIs which could be calculated using existing interRAI data. The interRAI assessment tools are copyrighted by interRAI and it is up to interRAI, as an organization, to decide whether or not certain assessments should be modified in the future. The first author is an interRAI Fellow, as are many co-authors, and will share our results with the broader interRAI organization for their consideration. However, our research team has no capacity to modify the existing instruments.*

5. It remains unclear whose quality is to be measured by the indicators – are specific providers accountable for the features measured? Or a regional health system as a whole? Please specify.

*We anticipate that the QIs can be used by multiple groups including individual health care organizations, consumers, and policy makers. We have added text in the discussion to make this more explicit (lines 402-406). We touch on this again in lines 426-432.*

6. Please specify what RN (line 114) means – research nurse?

*This term, RN, represent registered nurse and we have spelled this out in full.*

7. What exactly are “knowledge users” (line 160) and why is it important that they did the recruitment of patients and family members?

*In our project, “knowledge users” are typically health care administrators or directors who have an ability to use research results to inform decisions surrounding programs or policies. We have added some new text to explain that the participating organizations felt it was most appropriate to have KUs involved in recruiting patients/families. It was considered inappropriate, and potentially a violation of research ethics, for the research team to directly contact individuals receiving PC and their families (lines 161-163).*
