# Supplementary material for: A multi-stage process to develop quality indicators for community-based palliative care using interRAI data
Source: PLoS One. 2022 Apr 7;17(4):e0266569. doi: 10.1371/journal.pone.0266569 (PMC8989210; doi:10.1371/journal.pone.0266569)
Supplement: Attachment — Submitted filename: response to reviewers_mar322.docx [file pone.0266569.s005.docx]

**Reviewer #1:**

Following amendments reviewer concerns have been addressed. It is evident that manuscript authors have considered all comments to some extent. Although accepted, I would still be interested in seeing a clearer statement about ethics. Line 160 identifies KUs recruited those in receipt of PC and their families. Line 161 because it was inappropriate for the research team to do so. Line 165 identifies 8 KUs on the research team? I imagine KUs have stringent ethical probity however, this is not declared and lines 160, 161 and 165 appear to contradict each other.

*We apologize if this was confusing. The 8 KUs on our team acted as a “conduit” to families and individuals receiving palliative care and helped to link us to these individuals. The KUs and our team felt that it would be inappropriate for us, as a research team, to reach out directly to families/care recipients. Instead, the KUs helped to share our project with potential participants who could then contact us IF they were interested. For example, the KUs helped us by posting flyers on their web pages or by emailing study information to potential participants. In this sense, they were not participants themselves, but were simply helping us to identify individuals who were willing to take part in the research. We have modified line 161 and added one new sentence (line 164-165) to help clarify this process.*

**Reviewer #2:**

1. As I described, the generalizability of this results should be discussed. First of all, the author did not discuss about actual dataset of interRAI but interRAI data elements (e.g. data components) only. Thus, these indicators by authors are just only agreement of these elements could be useful for palliative care through consensus strategy. Therefore, the author should describe not only advantage of these indicators but also disadvantages such as lack of information compared with the previous literatures. The advantages of these indicators could be more contrasted. For example, “Prevalence of falls” is unique as indicators for palliative care, especially under sub-theme of symptom management.

*To clarify, the “interRAI dataset” is made up of the data elements within the assessment and nothing else, so the two things are identical. To be more explicit about which QIs have been cited in the literature, but which we could not measure, we have added references to the literature for those specific QIs where we mention them on lines 376-382 (please note that these added references do not show up in the “track changes” in the word document, but include ref #18, 63, 64 and 65).We feel that we have already described the unique advantages of the QIs derived using the interRAI dataset in the discussion section and feel it would make the discussion somewhat repetitious to add further comment on this.*

2. As far as I am correct, the panel members for this unique Delphi manuscript, especially for Phase III seemed to be completely different from Phase I and II. This should be clarified not only in Results section but also in Method section. As it has not still been improved, it could make readers confuse. The selection bias of panel members should also be described in limitation of this study.

*It was possible for decision makers participating in the Delphi panel to have also taken part in an interview/focus group during Phase I. We did not explicitly exclude them from the Delphi panel. We have modified lines 242-243 in the methods, and lines 291-292 in the results, to make this more explicit.*

*We do not feel that there was selection bias operating in the panel members. We had 6 individuals who consented, provided a demographic questionnaire but then did not complete the Delphi exercise. They were very similar to Delphi panel members in terms of age, gender, years of experience and clinical background. We have added additional text to describe this group (lines 294-298).*

3. The word about “domain” and “theme” could be used carefully not to make readers confuse in this manuscript. It would be better to describe how to determine these domains and themes by this study team (including all Phase I, II and III).

*We see these as two distinct concepts. We have used the term “domain” when discussing the QIs and the extent to which the indicators capture important information to assess quality. We found one instance where the term “domain” likely was inappropriate (line 329) and switched it to the word “area” to be more clear.*

*On the other hand, we have used the term “theme” specifically as it relates to the qualitative analysis of the workshop results, the focus groups, and interviews. We have added some text (line 153) to make it more clear with respect to the analysis of the workshop data. A quick search confirms that the term “theme” has always been used in this context in the manuscript. This is the accepted wording used in qualitative research to highlight a topic of discussion that was raised by multiple participants. It reflects the way in which the key issues raised by participants “emerge” during the qualitative analysis. As such, we feel that these two terms are indeed unique and have been used appropriately throughout the paper.*
